# Persistent hypercobalaminemia three months after successful gradual attenuation of extrahepatic shunts in dogs: a prospective cohort study

**DOI:** 10.1186/s12917-021-03123-1

**Published:** 2022-01-06

**Authors:** Nausikaa Devriendt, Gonçalo Serrano, Dominique Paepe, Sophie Vandenabeele, Emmelie Stock, Hilde de Rooster

**Affiliations:** 1 Small Animal Department, Faculty of Veterinary Medicine, Ghent University, Salisburylaan 133, 9820 Merelbeke, Belgium; 2 Department of Medical Imaging of Domestic Animals and Small Animal Orthopaedics, Faculty of Veterinary Medicine, Ghent University, Salisburylaan 133, 9820 Merelbeke, Belgium

**Keywords:** Canine, Vascular anomaly, Liver dysfunction, Vitamin

## Abstract

**Background:**

Deficiencies in vitamin A and D and disorders in the vitamin B complex are often present in people with chronic liver diseases. So far, the serum concentrations of these vitamins have not yet been studied in dogs with congenital extrahepatic portosystemic shunts (EHPSS), who also have some degree of liver dysfunction. The objective was to assess serum vitamin concentrations in dogs with EHPSS from diagnosis to complete closure. A prospective cohort study was performed using ten client-owned dogs with EHPSS, closed after gradual surgical attenuation. Serum concentrations of vitamin A, 25-hydroxyvitamin D, folic acid, cobalamin and methylmalonic acid (MMA) were measured at diagnosis prior to institution of medical therapy, prior to surgery, and three months after gradual attenuation and complete closure of the EHPSS.

**Results:**

At diagnosis, median serum concentrations of vitamin A, 25-hydroxyvitamin D and folic acid were 18.2 μg/dL (8.8 - 79.5 μg/dL), 51.8 ng/mL (19.4 - 109.0 ng/mL), and 8.1 μg/L (5.2 - 14.5 μg/L), respectively, which increased significantly postoperatively (88.3 μg/dL (51.6 - 182.2 μg/dL, *P*=0.005), 89.6 ng/mL (49.3 - >150.0 ng/mL, *P* =0.005), and 14.8 μg/L (11.5 - 17.7 μg/L, *P* <0.001), respectively). Median serum cobalamin concentrations were 735.5 ng/L (470 - 1388 ng/L) at diagnosis and did not significantly decrease postoperatively (*P* =0.122). Both at diagnosis and three months postoperatively 7/10 dogs had hypercobalaminemia.

**Conclusions:**

Serum concentrations of vitamin A, 25-hydroxyvitamin D and folic acid significantly increase after surgical attenuation. Nevertheless, persistent hypercobalaminemia is suggestive of ongoing liver dysfunction, despite successful surgery.

## Background

Vitamins are important micronutrients that are involved in a variety of physiological functions. The liver plays an important role in the digestion, absorption, storage and metabolism of vitamins, but also dietary uptake and intestinal absorption determine blood vitamin concentrations [1–3]. In humans with end-stage liver diseases, vitamin A and D deficiencies are often present [4]. Deficiencies in these fat-soluble vitamins are most likely secondary to malabsorption associated with end-stage liver failure [5]. Deficiencies in water-soluble vitamins can also occur in people with end-stage liver diseases, with deficiencies in the vitamin B complex more commonly seen in humans with alcoholic compared to non-alcoholic chronic liver diseases [6, 7]. However, more recently, hypercobalaminemia has also been associated with liver disease in people, cats and dogs [8–10].

Portosystemic shunts (PSS) are anomalous vessels connecting the portal system to the systemic circulation [11], causing hypoperfusion of the liver [12]. In an experimental study in dogs in which portocaval shunts were created and 65% of the liver was resected, hydroxylation of injected vitamin D into 25-hydroxyvitamin D was significantly lower than in dogs in which 65% of the liver was resected without shunt creation as well as in dogs that underwent a sham surgery. In this study, hepatic hypoperfusion had a greater impact on plasma concentrations of 25-hydroxyvitamin D than reduction in liver mass [13]. Experimental rats in which a portocaval shunt was experimentally created had a 25% decrease in serum vitamin A concentrations compared to sham-operated rats 48 days after surgery [14]. Besides hypoperfusion, some degree of (secondary) liver dysfunction is expected [15]. Rats in which portocaval shunts were created have a decreased concentration of cytochrome P450 due to hypoperfusion, causing liver dysfunction [16]. Data on vitamin concentrations in dogs with congenital PSS is sparse. One study analyzed vitamin C plasma concentrations in dogs with extrahepatic PSS (EHPSS). In dogs, vitamin C is synthesized by the liver, nevertheless only two out of 15 dogs had vitamin C concentrations under the lower reference value [17]. This can most likely be explained because of sufficient dietary uptake.

The current study aimed to assess serum concentrations of vitamin A, 25-hydroxyvitamin D, and molecules associated with the vitamin B complex in dogs with EHPSS before medical therapy, minimally four weeks after initiation of medical therapy, and three months after successful surgical attenuation as defined by transsplenic portal scintigraphy.

## Results

### Study sample

Initially, 15 dogs were included, of which five were excluded because of persistent shunting. The remaining 10 dogs consisted of different breeds: three cross breed dogs, two Pugs, and one each of Papillon, West Highland white terrier, Dachshund, Yorkshire terrier and Maltese. Median age at diagnosis was 12.5 months (3 - 105 months); five dogs were less than one year of age, with one of them being more than one year of age three months postoperatively. At diagnosis, the median body weight was 3.0 kg (1.4 - 7.8 kg) and the median BCS was 3/9 (2 - 6/9). All dogs presented with some degree of hepatic encephalopathy and one or more gastrointestinal signs, such as hyporexia, weight loss, vomiting and/or diarrhoea. Four dogs also presented with urinary signs, such as stranguria and hematuria. One dog had two EHPSSs which were attenuated during two separate surgeries, four months apart. Samples of this dog were analyzed at time of diagnosis, time of the first surgery and three months after the second surgery. The median time of medical therapy prior to surgical attenuation was 6 weeks (4 - 7 weeks), and the median time between surgery and the last follow-up visit was 14.5 weeks (13 - 31 weeks).

### Serum concentrations of vitamins and MMA

**Fig. 1:**
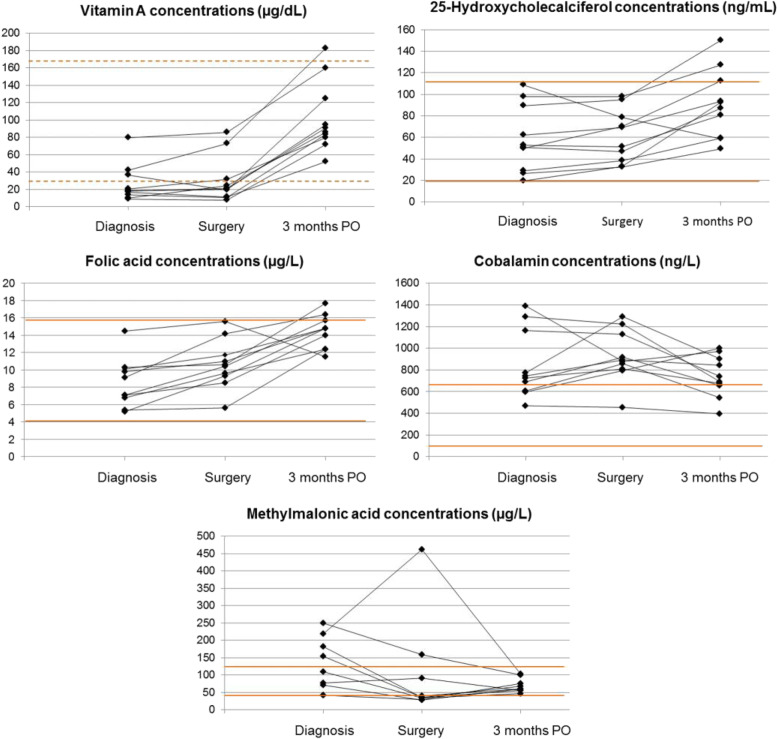
Serum concentrations of vitamin A, 25-hydroxyvitamin D, folic acid, cobalamin and methylmalonic acid in dogs with extrahepatic portosystemic shunts. Legend: 3 month PO – 3 months postoperatively. The upper limit of detection of 25-hydroxyvitamin D was 150.0 ng/mL

Samples were stored for a median time of 14 months (6-22 months). Serum concentrations of vitamins and MMA over time are depicted in Table 1 and Figure 1.

**Table 1:** Concentrations of different vitamins and methylmalonic acid in dogs with extrahepatic portosystemic shunts

	Diagnosis	Surgery*	Three monthspostoperatively*	Reference interval
	median (range)	median (range)	median (range)	
Vitamin A (μg/dL)	18.2	20.7	88.3‡°	30.6 – 169.6^[18]^
	(8.8 – 79.5)	(7.6 – 85.9)	(51.6 – 182.2)	
25-hydroxyvitamin D (ng/mL)	51.8	60.1	89.6	20.0 – 115.0
	(19.4 – 109.0)	(32.2 – 98.1)	(49.3 – >150.0)**	
Folic Acid (μg/L)	8.1	10.5	14.8‡°	4.0 – 16.0
	(5.2 -14.5)	(5.6 – 15.6)	(11.5 – 17.7)	
Cobalamin (ng/L)	736	885	715	99 – 658
	(470 – 1388)	(453 – 1290)	(394 – 999)	
Methylmalonic acid (μg/L)	93.6	37.4†	60.6	45.4 – 129.0
	(41.9 – 250.1)	(28.0 – 462.7)	(46.1 – 100.6)	

^*^Only data after the second surgery are reported of the dog with two single EHPSS that underwent two surgeries

^**^The upper limit of detection of 25-hydroxyvitamin D in the chemiluminescent microparticle immunoassay was 150.0 ng/mL

^[18]^published ranges of vitamin A concentrations in healthy dogs, also determined by reversed phase high performance liquid chromatography [18].

Statistically significant differences based on Kruskal-Wallis tests are indicated

†significant difference between median serum concentrations at diagnosis and time of surgery

‡significant difference between median serum concentrations at diagnosis and three months postoperatively

°significant difference between median serum concentrations at time of surgery and three months postoperatively

The serum vitamin A concentration was less than 30 μg/dL in seven dogs at time of diagnosis and in eight prior to surgery. Three months postoperatively, the serum vitamin A concentration was more than 90 μg/dL in five dogs. On an individual basis, serum vitamin A concentration increased significantly from diagnosis as well as from time of surgery to three months postoperatively (*P* =0.001 and *P* =0.005, respectively). Dogs less than one year of age had significantly lower serum vitamin A concentrations prior to surgery compared to dogs one year of age or older (*P* =0.032).

The serum concentration of 25-hydroxyvitamin D was only slightly below the lower reference limit in one dog at time of diagnosis. The 25-hydroxyvitamin D serum concentrations were within the reference interval for all dogs prior to surgery, whereas two dogs had 25-hydroxyvitamin D serum concentrations above the upper reference value three months postoperatively. A statistically significant increase in the 25-hydroxyvitamin D serum concentration was seen in individual dogs from diagnosis to three months postoperatively (*P* =0.005).

Folic acid serum concentrations were within reference interval in all dogs at time of diagnosis and prior to surgery. Three months postoperatively, only one dog had a serum concentration of folic acid slightly above the upper reference value. On an individual basis, folic acid serum concentrations significantly increased from diagnosis to three months postoperatively (*P* <0.001).

The serum concentrations of cobalamin were above the upper limit in seven dogs at diagnosis, nine dogs prior to surgery and in seven dogs three months postoperatively. One dog had normal cobalamin serum concentrations during all three measurements. Over time, no significant differences in serum concentrations were seen on an individual basis (*P* =0.122).

At time of diagnosis, the serum concentrations of MMA were below the lower reference value in two dogs and above the upper reference value in four dogs. Prior to surgery, seven dogs had MMA serum concentrations below the lower reference value, whereas it was above the upper reference value in one dog. A significant decrease in MMA concentration was found on an individual basis from time to diagnosis to time to surgery (*P* =0.042). Dogs less than one year of age had significantly lower MMA serum concentrations prior to surgery compared to dogs one year of age or older (*P* =0.008).

## Discussion

The current study found that the majority of dogs with EHPPS had low serum vitamin A concentrations and hypercobalaminemia at presentation, whereas serum MMA concentrations were variable at diagnosis. After starting medical therapy, serum MMA concentration significantly decreased. Over time, serum concentrations of vitamin A, 25-hydroxyvitamin D and folic acid increased significantly. In contrast, hypercobalaminemia persisted, despite successful surgery.

In the majority of dogs, serum vitamin A concentrations were low at diagnosis and prior to surgery, and serum concentrations increased two to eight times from surgery to three months postoperatively. This is in line with findings in experimental rats, in which a 25% decrease in serum vitamin A concentrations was seen after creation of a portocaval shunt compared to sham-operated rats [14]. Vitamin A, an essential fat-soluble vitamin is important in the regulation of cell differentiation, proliferation and apoptosis, in immune function, and it also regulates the homeostasis of carbohydrates, lipids and proteins [19–21]. The liver plays an important role in vitamin A uptake from chylomicrons, metabolism, and storage, with hepatocytes being the most important cell in the uptake and metabolism of vitamin A and with hepatic stellate cells being the major site of vitamin A storage [22]. Blood vitamin A is bound to retinol-binding protein (RBP), which is mainly produced by the liver [23]. The serum vitamin A concentrations at time of diagnosis and prior to surgery were lower than three months postoperatively in all dogs in the current study. The increase in serum concentration three months postoperatively can possibly be explained by increased production of RBP due to improved liver function. Of note, low serum concentrations of vitamin A do not necessarily mean that hepatic vitamin A concentrations are low, as rats in which a portocaval shunt has been created showed low serum vitamin A concentrations because of impaired release of vitamin A from the liver [14]. Further research assessing both serum and liver vitamin A concentrations together with RBP is needed to get more insight in the changes in the vitamin A concentrations observed in dogs with EHPSS. As sufficient vitamin A is very important during growth, oral supplementation can help to avoid growth retardation [24]. The latter is especially important as vitamin A serum concentrations were significantly lower in dogs less than one year of age at time of surgery, which indicates a relative shortage of vitamin A in the liver-support diet used in the current study for immature dogs. Although no studies are available specifically looking at changes of serum vitamin A concentrations in growing dogs, one study looking at the safety of vitamin A concentrations in food of growing dogs, revealed that retinol concentrations were significantly lower in dogs until 26 weeks of age [25]. In this study, three dogs were less than 26 weeks at diagnosis; although the increase three months postoperatively can be (partially) contributed to growing of these dogs, the increase in vitamin A concentrations in these dogs was not clearly more than in all other dogs.

In dogs, contrary to people, vitamin D is an essential fat-soluble vitamin [26]. It plays an important role in calcium homeostasis. Low blood concentrations are associated with inflammation, oncogenesis and cardiovascular disease. After intestinal absorption, vitamin D is transformed into 25-hydroxyvitamin D in the liver [26]. Whilst serum concentrations 25-hydroxyvitamin D remained within the reference interval in the majority of dogs at all time points, a significant increase was found over time. This is most likely attributed to increased transformation of vitamin D into 25-hydroxyvitamin D by the liver. A previous study, showed that lactulose at a dose of 1 g/kg/day causes an increase in calcium absorption in the intestines in healthy dogs [27]. Although the same dose was used in the current study, no clear influence of lactulose was observed, as no significant changes in serum concentrations of 25-hydroxyvitamin D were found after starting medical therapy. In small dog breeds, oral calcium supplementation causes an increase in plasma 25-hydroxyvitamin D, something which is not observed in large breed dogs [28].

This study showed that folate (vitamin B9) remained within normal limits, whereas cobalamin (vitamin B12) was above the upper reference value in the majority of dogs at all time points. Both cobalamin and folate are important vitamins in the synthesis of methionine, a non-essential amino acid. By catabolizing homocysteine into methionine, folate is transformed into folic acid [29]. Deficiencies in one of those vitamins can lead to decreased metabolism of homocysteine to methionine. Dietary folate is absorbed throughout the jejunum, after which is it taken up by the liver, where it is stored [30, 31]. Besides catabolism of methionine synthetase, folate is also responsible for metabolism of other amino acids such as cysteine, serine, glycine and histidine, and it also plays an important role in purine and pyrimidine nucleotide biosynthesis [31]. Folate undergoes an enterohepatic cycle which is important for the folate homeostasis [32]. In people, folate deficiencies are seen in people with several chronic liver diseases [33, 34]. Although serum folate concentrations remained within reference values, a significant increase was found between diagnosis and three months postoperatively. A possible explanation can be that untreated dogs have a decreased folate storage in the underdeveloped liver, which improves after surgical attenuation. To confirm this hypothesis, hepatic folate concentrations would need to be determined. In people, laxative treatment has been shown to increase plasma homocysteine concentrations and concurrently decrease folate concentrations [35]. In the current study, however, there are no indications that the dose of lactulose administered to decrease ammonia absorption from the intestinal tract did have a measurable effect on serum folate concentrations.

Cobalamin is a water-soluble vitamin which is mainly absorbed in the ileum and, in healthy dogs, cobalamin is mainly stored in the liver, and to a lesser extent, in the kidneys [36]. Similar to folate, the majority of cobalamin is recycled via the enterohepatic cycle [29]. Cobalamin is a key molecule that catalyzes metabolic reactions. Remarkably, in humans it is shown that clinical signs associated with hyper- or hypocobalaminemia, such as megaloblastic anemia and myeloneuropathy, are alike [8, 37]. In the current study, no clinical signs associated with hypercobalaminemia were observed in any of the dogs. For many decades, cobalamin deficiencies were associated with liver dysfunction [6, 7]; but, more recently, it has been documented that some people with liver disease have hypercobalaminemia instead [8]. Transcobalamins (TCB) bind serum cobalamins. Only 20% is bound to TCB II, which is the active fraction, whereas 80% of serum cobalamin is inactive and bound to TCB I and III [8]. Transcobalamin II is mainly synthesized by the liver, and once cobalamin is bound, it is taken up by the liver and other tissues and undergoes the enterohepatic cycle. In case of liver dysfunction, less TCB II is synthesized, increasing the concentrations of serum cobalamin bound to TCB I and III causing hypercobalaminemia and concurrent functional cobalamin deficits [8]. A retrospective study consisting of 654 dogs and 323 cats in which cobalamin was analyzed revealed that hypercobalaminemia is a rather rare condition; only 3% of dogs (76% were <10 kg) and 11% of cats. The majority of dogs and cats having gastrointestinal disease including hepatopathy, had increased serum cobalamin concentrations [10]. In an older study, 44 of 156 cats had hypercobalaminemia, of which the majority had either neoplasia or a hepatobiliary disease [9]. In the latter study, one cat with hypercobalaminemia was reported to have portal vein hypoplasia, which is a congenital disorder in which microscopic intrahepatic portovenous shunts are present [38]. Hypercobalaminemia seen in dogs with EHPSS could be explained by impaired hepatic uptake of cobalamin and by decreased TCB II synthesis. It is, however, surprising that, three months after surgery, hypercobalaminemia persisted in the majority of dogs, suggesting, at least, incomplete recovery of liver function despite successful surgery. Further research is needed to determine TCBs in dogs with EHPSS and to confirm this hypothesis.

In humans, MMA is used as a specific marker for cellular cobalamin deficiency and, therefore, it is commonly determined in patients suspected of having hypocobalaminemia [39]. Methylmalonic acid is formed during metabolism of several amino acids and odd chain fatty acids. Intracellular cobalamin deficiencies cause intracellular MMA accumulation which can subsequently cause methylmalonic acidemia [29]. Excess of MMA can inhibit carbomoyl phosphate synthetase, an enzyme involved in the urea cycle, which normally metabolizes ammonia into carbamoyl phosphate [29], potentially exacerbating hepatic encephalopathy. In the current study, four dogs had increased MMA concentrations at time of diagnosis. Surprisingly, in only two of those dogs, simultaneous hypercobalaminemia was present, suggesting a functional cobalamin deficit [29]. In a third dog, the combination of hypercobalaminemia and increased serum MMA was observed the day of surgery. Concentrations of MMA are very dependent on the diet [40]. This was confirmed in the current study. At diagnosis, the food that the dogs received was divers and so were the serum MMA concentrations. Prior to surgery, serum MMA concentrations were significantly lower compared to diagnosis, with the majority of dogs having MMA serum concentrations under the lower reference value. Moreover, prior to surgery, serum MMA concentrations were significantly lower in dogs less than one year of age. The low MMA serum concentrations can be explained by the relative low protein levels in the liver-support diet the dogs received (16% protein in kibbles, 6.5% in canned food). The MMA serum concentration was well within normal reference limits three months postoperatively, despite the fact that all dogs still strictly received the liver-support diet. The most likely explanation for these apparently contradictory findings is that concentrations of branched-chain amino acids (isoleucine and valine) increased after normalization of hyperammonemia by surgical closure of the EHPSS. Branched-chain amino acids are typically decreased in dogs with PSS, and are used as alternative pathway to detoxify ammonia in muscle tissue and in the brain in case of hyperammonemia [41–43].

This study has some limitations. The number of dogs included was low, which might have caused type II errors; nevertheless, clear trends could be identified. Because of the relatively short postoperative follow-up, it remains uncertain whether or not hypercobalaminemia will persist at long-term. Furthermore, dogs at diagnosis received different types of food, which makes interpretation of the baseline values difficult. Nevertheless, besides a large variation in serum MMA concentrations, the serum concentrations of the other metabolites analyzed did not seem to be greatly impacted by the type of food the dogs received. Serum samples were stored for a relatively long period of time before analysis in batch. No studies are available about the influence of long-term storage of dog serum on vitamin concentrations. Finally, for vitamin A, no reference intervals were available. Hence, for the vitamin A concentrations found in this study, trends are more reliable than exact values that are more difficult to correctly interpret. More research is needed to consolidate the findings of the current study and to assess the importance of these findings in dogs with EHPSS. Ideally, a case-control study should be performed, including age- and breed- matched control dogs that receive the same liver-support diet. Furthermore, analyzing vitamin concentrations in both the blood and liver would also be interesting. Finally, determination of vitamin concentrations in dogs with persistent shunting and in dogs receiving medical therapy for a long period of time would help to understand the influence of surgical attenuation versus long-term medical therapy.

## Conclusions

Serum vitamin A concentrations were low at diagnosis in the majority of dogs and only increased after successful surgical attenuation. Research is needed to investigate the effect of vitamin A supplementation in dogs with EHPSS. Dogs with EHPSS had hypercobalaminemia, which persisted despite successful surgical attenuation of the EHPSS. So far it is unclear if hypercobalaminemia will normalize over time and how long this would take.

## Materials and methods

### Animals

Client-owned dogs with EHPSS prospectively enrolled for this study. Dogs were eligible for inclusion if they did not receive any medication nor a liver-support diet at time of diagnosis and if owners opted for surgical attenuation of the EHPSS after a minimum of 4 weeks of medical management. All dogs were diagnosed with an EHPSS based on blood examination (complete blood count, serum biochemistry, serum bile acid concentrations and fasted ammonia) followed by medical imaging (abdominal ultrasonography, computed tomography or transsplenic portal scintigraphy). Medical management consisted of a liver-support diet (Royal Canin hepatic, Royal Canin, Zaventem, Belgium) offered in small quantities divided over the day combined with lactulose (0.5 mL/kg 3 times daily, adjusted to effect; Lactulose EG, Eurogenerics N.V., Brussels, Belgium) and metronidazole (7.5-10 mg/kg twice daily; Stomorgyl, Merial, Toulouse, France). Gradual attenuation of the EHPSS was performed with either an ameroid constrictor or thin film banding [44, 45], depending on the preference of the attending surgeon. The gradual attenuating device was always placed as close as possible to the systemic circulation.

Postoperatively, medical therapy was continued until one month postoperatively, after which lactulose and metronidazole were ceased. The liver-support diet was continued exclusively until the follow-up visit three months postoperatively. During this follow-up visit, a transsplenic portal scintigraphy was performed to determine shunt closure. Shunt fractions of <4.3% were considered normal [46]. Dogs with persistent shunting were excluded from the study.

### Sample collection, storage and analyses

Dogs were requested to be fasted for at least 12 hours. Blood samples were taken at time of diagnosis, prior to surgery and three months postoperatively. A total of 3.5 mL of blood was taken form a jugular vein and placed in a serum tube. After clotting, the serum tube was centrifuged at 3500g during 5 minutes. The serum was separated and stored at -80 °C until analysis. Concentrations of vitamin A, 25-hydroxyvitamin D, folic acid, cobalamin and methylmalonic acid (MMA) were analyzed in a commercial laboratory. Serum vitamin A was determined using reversed phase high performance liquid chromatography (Ultimate, ThermoFisher Scientific) and MMA was determined using a liquid chromatography with tandem mass spectrometry. Chemiluminescent microparticle assays (Architect I 2000, Abbott) were used to determine 25-hydroxyvitamin D (chemiluminescent microparticle immunoassay), folic acid (chemiluminescent microparticle folate binding protein assay) and cobalamin (chemiluminescent microparticle intrinsic factor assay). For all molecules but vitamin A, reference intervals of the laboratory were available. Consequently, published ranges of vitamin A concentrations in healthy dogs determined using a similar laboratory technique were used for reference (Table 1) [18].

### Statistical analysis

Statistical analyses were performed using SPSS Statistics 26 (IBM). Kruskal-Wallis tests were performed to evaluate median serum concentrations of the different molecules at different moments in time. Friedman two-way analyses were performed to evaluate serum concentrations of the different molecules within dogs over time. In case significant differences were found with Kruskal-Wallis tests or Friedman two-way analyses, pairwise comparisons with Bonferroni correction were performed. A *P* -value of <0.05 was considered significant.

## Data Availability

All data generated or analyzed during this study are included in this published article.

## Abbreviations

BCS: Body condition score
EHPSS: Extrahepatic portosystemic shunt
MMA: Methylmalonic acid
PSS: Portosystemic shunt
RBP: Retinol-binding protein
TCB: Transcobalamins
